# MyPainPal, a Novel mHealth App to Improve Pain in Patients With Advanced Cancer: Single-Arm Pilot Study

**DOI:** 10.2196/79942

**Published:** 2025-12-30

**Authors:** Desiree R Azizoddin, Michael Hassett, Kris-Ann S Anderson, Daniela Kessler, Alexi Wright, Madeline Gorra, Benjamin Kematick, Isaac Chua, Douglas Brandoff, Kate Lally, Lida Nabati, Susan MacIsaac, James A Tulsky, Andrea Enzinger

**Affiliations:** 1 Department of Supportive Oncology Dana-Farber Cancer Institute Boston, MA United States; 2 Department of Medicine Harvard Medical School Boston, MA United States; 3 Division of Population Sciences Dana-Farber Cancer Institute Boston, MA United States; 4 OU Health Boston, OK United States

**Keywords:** cancer, mHealth, mobile health, pain management, opioids, constipation, symptom management

## Abstract

**Background:**

Pain is common among patients with advanced cancer and is often inadequately controlled. Opioids are central to treatment; yet, self-management is challenging, and clinicians lack scalable tools to monitor and support patients between visits.

**Objective:**

This study aimed to evaluate the feasibility and acceptability of MyPainPal (Dana-Faber Cancer Institute), a novel mobile health app designed to optimize cancer pain management. MyPainPal combines daily surveys assessing symptoms and analgesic use, algorithmic self-management support, tailored psychoeducation, and clinician monitoring. Secondary objectives were to explore preliminary clinical impact and identify priorities for refinement.

**Methods:**

This single-arm pilot study enrolled adults with advanced malignancies using opioids for moderate-to-severe pain from an outpatient palliative care clinic at a comprehensive cancer center. Participants used MyPainPal for 28 days while nurses monitored symptom responses via a secure portal, and also completed structured surveys at end-of-study. Primary assessment of usability and acceptability included the System Usability Scale (SUS; range 0-100), the Acceptability E-Scale (range 6-30), and ratings of satisfaction using a 5-point Likert scale. Semistructured debriefing interviews explored user experience, perceived impact, and suggestions for optimization.

**Results:**

Twenty participants with advanced cancer enrolled, with a mean age of 57 (SD 12.3) years, 55% (11/20) female, 80% (16/20) non-Hispanic White, with mixed cancer types. Over the 28-day study, patients logged into MyPainPal a median of 14 (IQR 8-17) times, and completed a median of 8 (IQR 5-14) symptom surveys, reflecting mean of 36% (SD 20%) of eligible (out-of-hospital) days on study. Usability and acceptability ratings of MyPainPal were high (mean SUS 78.3, SD 16.2; mean Acceptability E-Scale 24.0, SD 4.4); 79% rated overall satisfaction of greater than or equal to 4/5. Twenty percent of surveys generated an alert, prompting nurse outreach. In response, 5 participants had symptom medications changed and 2 had medication errors corrected. In debriefing interviews, many participants described that the intervention reduced barriers to pain reporting and facilitated timely and constructive interactions with care teams for symptom management. Several noted that the intervention validated their pain experience, reduced stigma around opioid use, enabled constructive conversations with providers, and promoted self-management. Patients recommended several survey modifications, including reducing their frequency and enabling more nuanced pain assessments. Participants underused the educational resources and suggested that they be featured more prominently. Some patients suggested that the MyPainPal app should be introduced earlier in patients’ cancer pain trajectory when pain needs are higher and opioid management is novel.

**Conclusions:**

In this pilot study, MyPainPal demonstrated feasibility, acceptability, and preliminary evidence of potential clinical impact among patients with advanced cancer receiving palliative care. The app has been rebuilt and optimized with attention to patient feedback and in preparation for a future efficacy study.

**Trial Registration:**

ClinicalTrials.gov NCT03717402; https://clinicaltrials.gov/study/NCT03717402

## Introduction

Pain is a common and distressing complication of cancer, affecting approximately two-thirds of patients with advanced-stage cancers [1,2]. Nearly 60% of these individuals experience moderate to severe pain [1,3], for whom opioids are recommended [4]. Yet despite access to opioids and other analgesics, many patients with cancer experience inadequate pain relief [3]. Poorly controlled pain degrades patients’ physical, functional, and emotional well-being [3] and is a leading cause of acute care use [5,6], underscoring the need to bolster outpatient pain management.

Barriers to effective pain management exist at the patient, provider, and health system levels. Patients underreport pain due to stoicism, opioid stigma, and a desire to avoid being distracted from cancer care [7]. Without reliable tools to track their symptoms, analgesic use, and side effects, patients often struggle to convey information that care teams need to optimize pain management. Moreover, patients’ ability to self-manage is hindered by knowledge gaps, including how best to use and sequence various analgesics and to manage side effects [8]. Opioid side effects are incredibly common, complex for patients to manage, and a frequent reason that patients undertreat their pain [9], particularly when compounded by fears of addiction [10-12]. Despite this complexity, opioids are typically prescribed without structured support or contact between clinic visits—creating ample opportunity for confusion, medication mismanagement, and unnecessary suffering.

Scalable interventions are needed to optimize the outpatient management of cancer pain. There is growing evidence that electronic assessment of patient-reported outcomes (PROs) can improve symptom management and reduce acute care use [13,14]. While increasingly developing, PRO interventions for cancer pain are generally underdeveloped and not integrated with patients’ clinical care [15-18]. Mobile health (mHealth) technology is an appealing strategy to deliver pain management support in patients’ home environments, as more than 85% of adults in the United States own a smartphone [19]. Our goal was to develop an mHealth intervention to optimize the outpatient management of cancer pain by combining patients’ self-reported pain symptoms, medication use, and side effects with timely advice and education, while concurrently documenting and relaying information to care teams. Here we report on a single-arm pilot study to evaluate the feasibility and acceptability of MyPainPal conducted with patients with advanced cancer as they manage chronic cancer pain using opioids.

## Methods

### Study Overview

**Figure 1:**
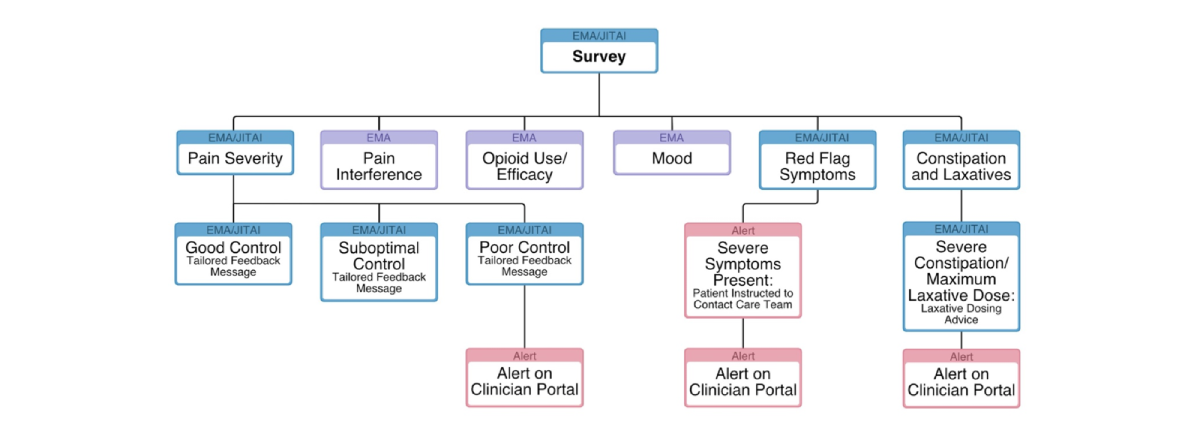
Ecological Momentary Assessments and Just-In-Time Adaptive Interventions for short (daily) surveys in the MyPainPal app for patients with advanced cancer managing pain. EMA: Ecological Momentary Assessments; JITAI: Just-in-Time Adaptive Intervention.

**Figure 2:**
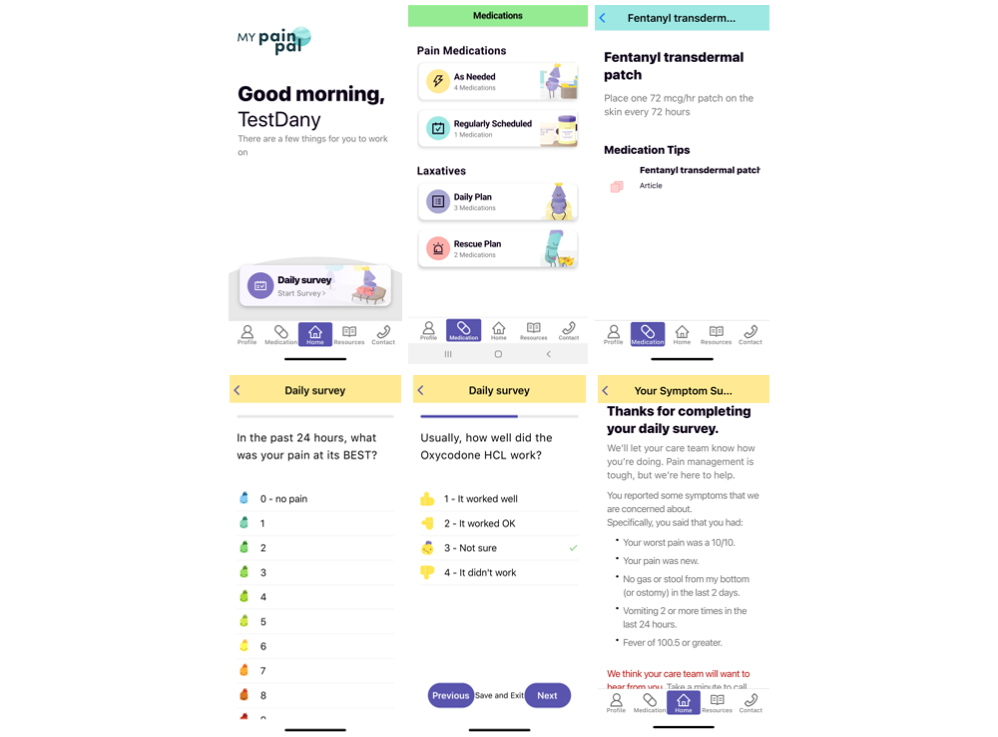
Screenshots of the MyPainPal mobile health app that delivers pain and symptom support to patients with advanced cancer using opioids to manage pain.

This was a single-arm pilot feasibility study of the MyPainPal mHealth app (NCT03717402, registration date: October 10, 2018). Our interdisciplinary research team developed MyPainPal using an iterative process combining the Agile [20] and mHealth Development and Evaluation Frameworks [21] that we previously described [22]. The intervention consists of a patient-facing smartphone app featuring a library of comprehensive cancer pain education and a “virtual medicine cabinet” that hosts patients’ specific analgesic and laxative medications. Daily surveys deliver tailored symptom support (Figures 1 and 2). MyPainPal also offers a HIPAA (Health Insurance Portability and Accountability Act)-compliant web portal tailored for providers.

In Figure 1, daily symptom and medication use surveys were pushed at 10 AM each calendar day and were available for completion until midnight. The Ecological Momentary Assessments (EMAs) drew from the Brief Pain Inventory [23] to assess pain severity and interference in the past 24 hours, with adaptive survey logic for “red flag” symptoms (eg, new or worsening chest pain, fever, severe vomiting, and symptoms of bowel obstruction) when patients reported severe pain (>7 on Numeric Rating Scale [NRS]). Including the name and dose of patients’ specific short-acting opioid, they indicated doses taken, degree of pain relief, and whether their pain control was acceptable in the past 24 hours. An additional 4 items from the Positive and Negative Affect Schedule (PANAS) scale [24] assessed mood in the past 24 hours. To enable self-management support for opioid-induced constipation, patients indicated the number of days since their last bowel movement, stool characteristics, and laxative doses used in the past 24 hours. Once per week, the survey included additional items assessing use of other pain medications, opioid side effects, and psychological factors, including pain catastrophizing [25] and sleep quality [26]. Just-in-Time Adaptive Intervention (JITAI) technology provided patient-facing feedback in response to symptoms on a summary screen presented immediately following survey completion.

In Figure 2, from left on the first row, there is a screenshot of the home screen, the Medication cabinet, and examples of medications. In the second row, there are screenshots of pain ratings, opioid medication reporting, and end-of-survey summary feedback that follow survey completion.

### MyPainPal Intervention Overview

EMAs were pushed at 10 AM each day, assessing symptoms and medication use over the past 24 hours. The EMAs drew from the Brief Pain Inventory [23] to assess pain severity and interference (0-10, NRS); adaptive survey logic assessed for “red flag” symptoms (eg, new or worsening chest pain, fever) if participants rated their pain severity 7 or higher. Surveys included participants’ prescribed opioid medications, and they reported the number of doses taken in the past 24 hours, the degree of pain relief, and whether their pain control was acceptable. An additional 4 items from the PANAS scale [24] assessed mood. To enable self-management support for opioid-induced constipation, participants reported constipation severity, days since their last bowel movement, and doses of laxatives taken. Once per week, a slightly longer version of the survey also assessed use of other pain medications, side effects, pain catastrophizing [25], and sleep quality [26].

MyPainPal uses JITAI technology to tailor feedback in response to pain and constipation reporting (Figure 1). Algorithms based on pain severity and pain acceptability categorize patients’ responses into good, fair, and poor pain control [22]. Drawing from message banks tailored to each of these conditions, a summary screen (Figure 2) presents a brief, empathic statement, an educational “pearl,” and links to relevant content. For constipation, the summary screen includes laxative dosing suggestions based on days since last bowel movement and doses of senna and polyethylene glycol taken in the preceding 24 hours, adapted from clinical decision support algorithms developed by Yackel et al [27]. The summary screen additionally instructs participants to contact their care team for poorly controlled pain or constipation, or for any severe symptoms (eg, pain severity >9/10, any “red flag” symptoms). A HIPAA-compliant web portal displays participants’ symptom reports and medications, and includes a dashboard to facilitate triage and participant management.

### Setting and Participants

The study was conducted in the outpatient palliative care clinic of the Dana-Farber Cancer Institute (DFCI), a comprehensive cancer center in Boston, MA. English-proficient adults were eligible if they had an advanced solid malignancy or multiple myeloma, were being treated with palliative intent, had chronic pain related to their cancer or cancer treatment, reported an average pain severity of >4/10 (NRS), took opioid pain medications, and owned a compatible smartphone (iOS or Android). Exclusion criteria included cognitive impairment, history of opioid use disorder, recent bowel obstruction, hospice enrollment, or use of buprenorphine (not app supported).

### Recruitment and Study Procedures

Participants were recruited between June and October 2021 and from February through April 2022, after pausing recruitment to correct an app bug. Potentially eligible patients were identified by screening clinic schedules or direct referrals. After receiving permission from the treating clinician, a research assistant approached patients for participation in person or by phone and obtained written informed consent [28]. After participants completed a baseline survey, study staff conducted an onboarding visit over video (Zoom; Zoom Video Communications) or telephone during which they helped participants download the app and conducted a brief tutorial. Patients were encouraged to complete the app’s daily symptom survey and use other app features. Our goal was to allow patients flexibility to use the program in the way that seemed most useful to them, rather than mandate a particular level of engagement. Participants were reminded that the dashboard was not monitored 24/7 and to contact their care team for any severe symptoms.

Participants were on-study for 28 days and received 2 protocol-directed outreach calls 1 week after onboarding. First, the research assistant assessed and provided support for any technical difficulties. Second, a palliative care clinic nurse reviewed participants’ pain self-management challenges and provided advice. Thereafter, nurses monitored the portal on weekdays. The research assistant also sent the care team a symptom “alert” summary via the electronic health record (EHR) for poorly controlled pain, constipation, or any “red flag” symptoms. Nurses were advised to contact patients using their clinical discretion and to document triage phone calls in the EHR using a study template.

### Study Assessments

Patients completed surveys at baseline and end-of-study using REDCap (Research Electronic Data Capture; Vanderbilt University) [28]. Clinical factors were abstracted from the EHR. At baseline, patients self-reported demographics and completed the 5-item Media and Technology Usage and Attitudes Scale [29]. In an optional semistructured debriefing interview conducted over video (Zoom) or phone conducted within 4 weeks of completing their MyPainPal testing, a trained interviewer not personally acquainted with the participant (DRA and AE) assessed patients’ experiences using the app, features they appreciated and disliked, perceived impacts on symptom self-management, and recommended changes or features they disliked (refer to Multimedia Appendix 1 for interview guide).

### Measures

#### Feasibility and Acceptability Outcomes

Measures of feasibility included frequency of MyPainPal use and symptom survey completion. We did not predefine benchmarks for feasibility and acceptability, given lack of consensus regarding meaningful thresholds in app-based studies. At end-of-study, patients completed the validated 6-item Acceptability E-Scale [30], our primary measure of acceptability. Items were scored on a 5-point Likert scale (perceived ease, understandability, enjoyment, helpfulness, time spent, and overall satisfaction of the app) with scores ranging from 6 to 30 and higher scores being better. The validated 10-item System Usability Scale (SUS; range 0-100) assessed usability, relying on the standard benchmark of >68/100 [31].

#### Exploratory Clinical Outcomes

To explore clinical impact, research assistants tracked the number of symptom surveys completed in the app and symptom “alerts” triggered for severe symptoms. Using a codebook created by MG, DRA, and AE, structured chart abstraction assessed clinical outreach triggered by alerts, including documented phone calls and clinical actions, including medication counseling, medication changes, or advising an urgent clinical evaluation.

#### Exploratory Pain Outcomes

At baseline and end-of-study, patients completed the 15-item Brief Pain Inventory-Short Form [23], which measures pain severity and interference, the 13-item Pain Catastrophizing Scale that measures negative thoughts about pain (score 0-52) [25], the constipation items from the PRO-CTCAE [32], and the 27-item FACT-G [33] which assesses aspects of well-being (score 0-108).

#### Analysis

Simple frequencies and descriptive statistics assessed measures of feasibility, acceptability, and satisfaction. We explored differences between pre- and postintervention symptoms using paired-sample *t* tests. All analyses are complete case analyses, without imputation of missing data, and were conducted using IBM SPSS (version 28.0).

End-of-study interviews were transcribed verbatim and reviewed by a master’s-level research technician (KASA) with input from the primary investigators (DRA and AE). Codes were initially derived from the interview guide. Focusing on semantic meaning, KASA modified the codebook through simultaneous inductive coding and app of the deductive codebook driven by the interview guide. When new codes were identified that emerged naturally during interviews, in addition to those identified through the interview guide, KASA, DRA, and AE had a discussion to confirm additional codes, then KASA assimilated new codes into the codebook and recoded previous interviews. KASA, DRA, and AE identified preliminary topics, their component codes, and exemplar quotes. KASA, DRA, and AE combined and refined topic summaries between participants.

### Ethical Considerations

Study procedures were approved by the Dana-Farber and Harvard Cancer Center Institutional Review Board (IRB# 18-504) and conducted in accordance with the 1964 Declaration of Helsinki and later amendments. All participants provided written informed consent before trial participation. All data have been deidentified and remain anonymous. Upon consent, participants were provided a numerical identifier which was paired with their data thereafter. Participants received a US $50 gift card for participation.

## Results

### Study Participants

**Figure 3:**
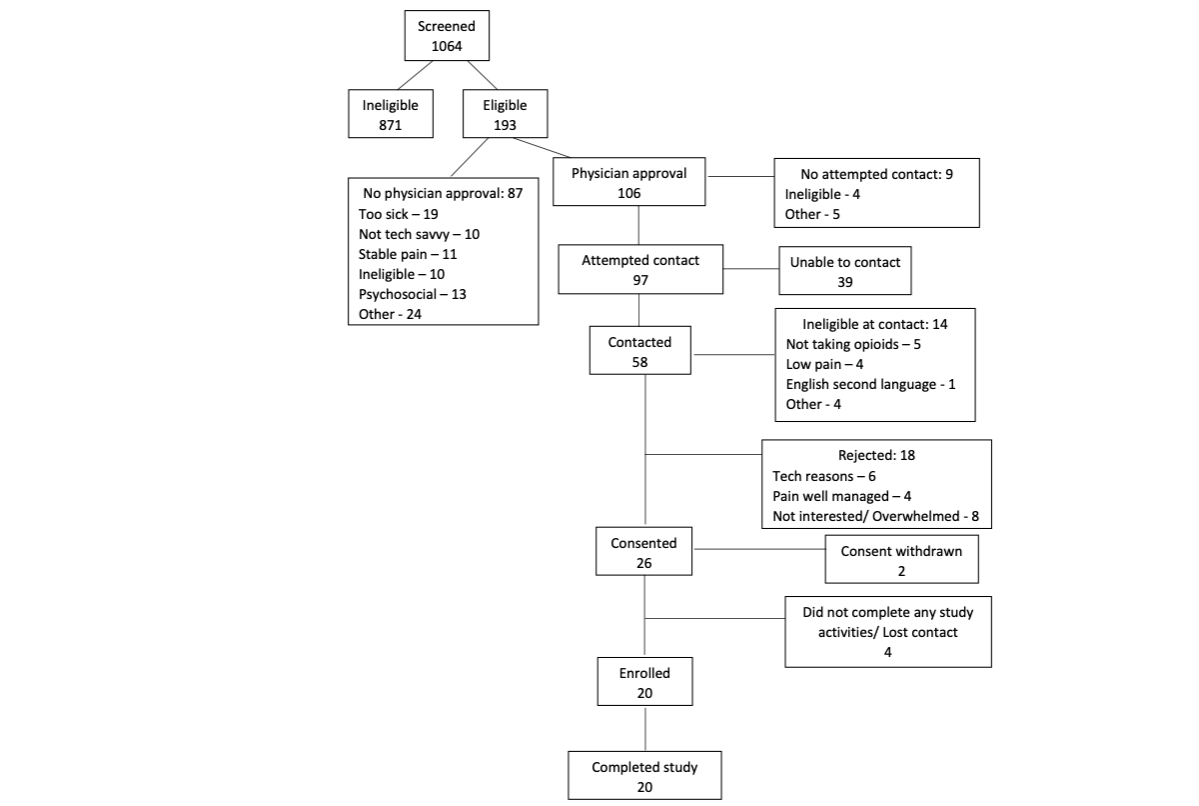
CONSORT (Consolidated Standards of Reporting Trials) diagram of MyPainPal single-arm pilot study in patients with advanced cancer managing pain recruited from outpatient palliative care.

Of 193 patients identified on initial screening, we received permission for and contacted 61, of whom 47 were eligible, 26 consented, and 20 enrolled in the study. Common reasons patients declined participation included technology challenges (eg, lack of comfort with smartphones), having stable pain, being overwhelmed or not being interested in participation. All 20 participants completed baseline and end-of-study surveys (Figure 3). Participants had a mean age of 57 (SD 12.3) years; 55% (11/20) were female; and most were non-Hispanic White (16/20, 80%; Table 1).

The most common cancers were breast and colorectal; 90% (18/20) of patients were on active cancer treatment; all patients were taking short-acting opioids and 75% (15/20) were taking long-acting opioids. Five (25%) patients were hospitalized during the study, ranging from 1 to 15 days.

**Table 1:** Participant characteristics of the MyPainPal single-arm pilot study (N=20)^a^.

Characteristics			Values
**Demographics**			
	Female, sex, n (%)		11 (55)
	Age (years), mean (SD)		57 (12.3)
	**Race, n (%)**		
		Non-Hispanic White	16 (80)
		Asian	2 (10)
		Black	2 (10)
	Marital status (married), n (%)		17 (85)
	Education (college degree or higher), n (%)		10 (50)
	Extremely confident filling out medical forms, n (%)		10 (50)
**Clinical characteristics**			
	**Cancer type, n (%)**		
		Breast	4 (20)
		Colorectal	3 (15)
		Prostate	2 (10)
		Lung	2 (10)
		Pancreas	2 (10)
		Other	7 (35)
	Receiving active cancer treatment, n (%)		18 (90)
**Opioid medications**			
	**Long-acting opioids, n (%)**		
		Morphine ER^b^	6 (30)
		Oxycodone ER	4 (20)
		Methadone	3 (15)
		Fentanyl transdermal patch	2 (10)
	**Short-acting opioids, n (%)**		
		Oxycodone IR^c^	15 (75)
		Hydromorphone	4 (20)
		Morphine IR	1 (5)
**Known pain syndromes at baseline, n (%)**			
	Malignant spinal cord compression		5 (25)
	Painful bone metastases		12 (60)
	Pathologic fracture		6 (30)
**Baseline pain characteristics, mean (SD)**			
	BPI^d^ worst (0-10)		5.85 (2.4)
	BPI average (0-10)		4.42 (1.8)
	BPI best (0-10)		3.00 (2.2)
	BPI pain severity (0-10)		4.35 (1.9)
	BPI interference (0-100)		35.5 (14.8)
	General activity (0-10)		5.45 (2.9)
	Mood (0-10)		4.80 (2.4)
	Walking (0-10)		5.35 (2.6)
	Normal work (0-10)		6.30 (3.0)
	Relationships (0-10)		3.15 (2.2)
	Sleep (0-10)		4.40 (2.6)
	Enjoyment of life (0-10)		6.05 (2.8)
**Technology use at baseline (** **>** **several times per month), n (%)**			
	Read emails		17 (80)
	Take pictures		16 (84)
	Check news		15 (79)
	Use apps		17 (90)
	Search information		17 (90x)

^a^BPI pain severity is a mean score (worst, average, best, and now). Technology use scores: never, once a month, several times a month, several times a week, once a day, several times a day, once an hour, all the time were recoded as a binary variable between once a month or less and >several times a month.

^b^ER: extended release.

^c^IR: immediate release.

^d^BPI: Brief Pain Inventory-Short Form.

### Feasibility and Acceptability

Patients logged into the app a median of 14 (IQR 9-17) times and completed a median of 8 EMAs (IQR 5-14) over the 28-day study. The mean rate of symptom survey completion was 34% (SD 21%; mean 9.40, SD 5.5) as a function of days on study, and 36% (SD 20%) as a function of eligible days (ie, out-of-hospital). A total of 188 symptom surveys were completed.

Of the 19 participants who completed usability and acceptability measures (one did not use the app and omitted these items), 79% (15/19) rated their overall satisfaction as greater than or equal to 4/5. The mean Acceptability E-scale score was 24 (SD 4.4), the mean SUS score was 78.3 (SD 16.2), meeting our usability benchmark of 68/100, and 65% (13/19) were likely or very likely to recommend the app to a friend (Table 2).

**Table 2:** Acceptability ratings of the MyPainPal app pilot study in patients with advanced cancer managing pain^a^.

Acceptability items	Mean (SD)	Percent rating >4 out of 5, n (%) (N=19)
How easy was the MyPainPal app for you to use?	4.4 (0.9)	16 (84)
How understandable was the content in the MyPainPal app?	4.6 (0.7)	17 (89)
How much did you enjoy using the MyPainPal app?	3.5 (0.9)	7 (37)
How helpful was the MyPainPal app as you managed your pain?	3.3 (0.7)	6 (32)
How acceptable was the amount of time it took you to use the MyPainPal app?	4.2 (0.9)	14 (74)
How would you rate your overall satisfaction with the MyPainPal app?	4 (0.8)	15 (79)

^a^Acceptability E-scale modified for MyPainPal. Items were scored on a 5-point Likert scale, with higher scores representing greater acceptability.

### App Use and Assessment of Clinical Impact

Nineteen percent of symptom surveys (36/188 total) triggered an alert (Table 2) across 16 individual patients, with a median of 2 (IQR 1-2) alerts per patient. The most common symptoms triggering the alerts were pain (78%), red flag symptoms (25%), and constipation (14%). Nurses called patients all alerts, with some resulting in ≥1 call. Analysis of EHR documentation found that nearly all (97%) triage calls involved medication counseling, most frequently centered on optimizing the timing of medication use (eg, using analgesics before going to sleep or sequencing medications differently), and encouraging patients to take short-acting opioids more frequently or at the higher prescribed range. Triage calls directly resulted in prescription medication changes for 5 unique patients (25%), with 2 patients having >1 medication change. Additionally, the nurse calls identified and corrected errors in medication self-administration for 2 patients.

### Exploratory Outcomes

Although we did not test for pre-post differences in this exploratory, single-arm study, pain severity, pain interference, pain catastrophizing, physical well-being, social and familial well-being, functional well-being, and constipation ratings remained relatively stable from baseline to end-of-study. We observed slight decrements in emotional well-being and quality of life, which is unsurprising for this seriously ill population (Table S1 in Multimedia Appendix 2).

### Qualitative Patient Feedback

Fourteen of the 20 (70%) participants participated in optional debriefing interviews lasting between 15 and 47 minutes. Dominant themes were (1) aspects of the app that patients appreciated and used most, (2) dislikes and suggested improvements, (3) perceived effects of the app, and (4) perspectives on the optimal conditions for app use (Table 3).

**Table 3:** Themes and Representative quotes from optional poststudy qualitative interviews reviewing the MyPainPal app with patients with advanced cancer pain following testing for 4 weeks.

Themes and key subthemes		Representative quotes
**1. Aspects of the app that patients appreciated**		
	Easy to use	“I think the app was very straightforward…I thought it was really easy to use. I liked that” (60- to 70-year-old, male, colorectal cancer)
	Informative	“Yeah. And the resources are great….the like test your knowledge thing which was really cute. I actually did that this morning. And I thought that was pretty helpful just to drive – like drive home those, yeah. I thought that was pretty cool.” (40- to 50-year-old, female, breast cancer)
	Reinforced self-management	“Especially like the questions about…bowel movements which have been more regular for me. But that was really great because some days I would think, how long has it been? So doing this I took note every night so I knew… So that was good.” (40- to 50-year-old, female, breast cancer)
	Facilitated connection to care team	“This is the part I liked. I know the information I entered, the care team will see it.” (60- to 70-year-old, male, colorectal cancer)
	Symptom survey understandable and appropriate	“The questions were very straightforward, even though the pain questions you never—as a patient, you’re always hard—it’s hard to put numbers on pain…but I thought they were worded pretty well and were—I mean it was just easy. It was straightforward.” (40- to 50-year-old, male, colorectal cancer)
**2. Dislikes or recommended changes**		
	Survey-related issues	“You get the alert like at, what, 10:00 AM, or something like that, and then that’s not really the time that I was ready or able to do it.” (40- to 50-year-old, male, colorectal cancer) “So some of the questions, I believe they were kind of repetitive.” (70- to 80-year-old, male, renal cancer) “No, that was the biggest thing with me. When I was always done with it, I was like they should have a box where I could write where I’m hurting.” (30- to 40-year-old, female, glioblastoma)
	Features not used or noticed	“The screen at the end, we never looked at the summary. We were just submitting the survey, but we didn’t look at it.” (70- to 80-year-old, male, renal cancer) “Never knew that existed. Now the first time I saw anything about detailed constipation was yesterday” (60- to 70-year-old, female, pancreatic cancer)
**3. Perceived effects of the app**		
	Validating and comforting	“If you don’t have something to bridge the gap between you and the physician or doctor that you’re dealing with, you’re in no man’s land….my God, it’s pretty terrifying if you don’t have it….listen, if there was one thing it did, right, how would I describe it? It [the app] was my friend.” (60- to 70-year-old, female, gallbladder cancer)
	Informed and reinforced learning	“I found them [resources] interesting um they had a lot of information that I already knew. But then you know there were a couple of new things and even for the ones I already knew, it was a good reinforcement.” (70- to 80-year-old, female, multiple myeloma)
	Medication tracking	“It made me conscious of the fact that I ought to take the oxycontin at 3 o’clock in the afternoon and not be so cavalier about forgetting it, to the extent that I now travel with some in my purse.” (70- to 80-year-old, female, breast cancer)
	Lessened opioid stigma	“It sort of helped me with the stigma of it. Because I do feel really—you know, it’s really strange being on all these medications. We got kids, I’m trying to run around, I can’t run around like I used to. So I just have, there’s a bit—you know, that’s really heavy on me.” (40- to 50-year-old, female, breast cancer)
	Helped build insight into pain experience	“Yeah, I liked that [the app] made me think things through because [you] just get through the day, and then you know, days would go by and I would think, oh, I’m feeling worse, or how long have I been feeling like that? And since I started with the app, I was much more in tune. So that was very helpful.” (40- to 50-year-old, female, breast cancer) “…made me very conscious of the pain and all those… You know, was I irritable? Was I nervous? Was I…Pain has a lot of ramifications besides hurting.” (70- to 80-year-old, female, breast cancer)
	Facilitated care team interactions	“I’m not big on calling. I felt like I was bugging them. So, it was, I think for myself, it was good that the information was relayed, and then they would call me… There was a period there where I was talking to my care team every other day, or every few days [about changes in opioid use]. And that probably wouldn’t have happened otherwise. I probably would have only spoken with them when I came in to see them for my check-ups.” (30- to 40-year-old, male, lung cancer) “Yea. I think I would say I probably wouldn’t be as tempted to go to the emergency department knowing they would be reaching out to me.” (30- to 40-year-old, male, lung cancer) “I specifically remember [caregiver and patient] were downstairs in the living room chatting about—you know, ‘should we call, do we not call? You know it seems like it’s getting worse.’ And then [the nurse] called and it was like, she kinda took the burden off of us, you know?...And then she basically go the ball rolling with everything [medication changes].” (60- to 70-year-old, male, urothelial cancer)
**4. Optimal context**		
	Deliver earlier in cancer context Enable caregiver access Support existing self-management systems	“I’ve been doing relaxation exercises for a long time…but I think they’d be particularly helpful for people who are just beginning to use it.” (70- to 80-year-old, female, multiple myeloma) “I just keep it [a log of pain medications] in the spreadsheet. So did that and a couple time sent it off through the gateway.” (60- to 70-year-old, non-Hispanic White, urothelial cancer) “Especially if somebody didn’t have as good a background as I did already on the medications. It would be even more helpful. So, I think for somebody that didn’t already have a baseline of information, it would be extremely helpful.” (30-40 year-old, male, lung)

#### Themes 1 and 2 (Likes and Dislikes)

Many patients commented that the app was easy to use, informative, and educational. For example, a 40- to 50-year-old woman with breast cancer stated, “This app actually had everything I needed sort of right there. So that was really cool.” The most used feature was the symptom survey, which patients reported was easily understandable and of an appropriate length. Conversely, a few patients disliked the repetitive nature of the survey and suggested sending them less frequently or including more nuanced symptom assessments or options for free-text responses. Although the app sent survey reminders at 10 AM, many patients suggested making the timing customizable. Several patients also appreciated the app’s visual appeal and the use of animated characters.

Patients underused several app features, particularly the resource library and medicine cabinet. This may be because patients described only engaging with app features in response to notifications, which were only sent for symptom surveys. In contrast, other features were designed for patients to explore without any notifications to prompt use. A few patients expressed a desire to receive more specific feedback around symptoms; for example, suggesting different doses of pain medications or providing options for behavioral strategies.

#### Theme 3 (Perceived Effects of the App)

Patients shared that the app’s dominant impact was the facilitation of constructive interactions with the care team. Many patients expressed that the app lowered barriers to reporting their symptoms, provided comfort and reassurance because participants knew their symptom reports would be reviewed, and resulted in timely outreach from care teams with proactive counseling and medication changes in response. For example, a 70- to 80-year-old woman with multiple myeloma stated,

...There was really good communication with my care team so in that way it was helpful. You know I might’ve been a little shy about saying that this pain has gotten really horrible but you know using the app and quantifying it was it was easier that it was reported that way.

Another 30- to 40-year-old male with lung cancer explained,

...I’m not big on calling. I felt like I was bugging them. So, I think for myself, it was good that the information was relayed, and then they would call me… There was a period there where I was talking to my care team every other day, or every few days [about changes in opioid use]. And that probably wouldn’t have happened otherwise.

Several patients described the app as a source of comfort, reassurance, or validation. One patient stated, “I mean, listen, if there was one thing it did, right, how would I describe it? It was my friend,” (60- to 70-year-old, female, gallbladder cancer).

Many patients conveyed that the app helped them acquire or consolidate learning about symptom management, gain new insights into their symptoms, and facilitate better medication use and tracking. One described how daily EMAs helped to bolster insights about her pain experience:

...*The questions – I liked that they made me think things through because [you] just get through the day, and then you know, days would go by and I would think, oh, I'm feeling worse, or how long have I been feeling like that? And since I started with the app, I was much more in tune. So that was very helpful.* (40- to 50-year-old, female, breast cancer).

Many described that reporting opioid use and reviewing opioid education not only increased their knowledge but also helped to reduce the stigma surrounding opioids, which enabled them to feel more comfortable using opioids when needed. A 70- to 80-year-old woman with breast cancer described that the app *“made me conscious of the fact that I ought to take the oxycontin in the afternoon and not be so cavalier about forgetting it to the extent that I now travel with some so that I don’t forget it.”* A few patients relied on the app for laxative titration instructions; however, many patients did not notice this advice as it required scrolling down to the end of the symptom summary screen. In contrast, a few patients described that they were already knowledgeable about the education available in the app and that it did not help them to build new pain insights.

The final theme was the optimal context for the app to have the most impact and thoughts on barriers to use. Several believed the app would be more beneficial for patients earlier in their disease course or for those with more severe or dynamic pain, since they described having received this education from their palliative care providers in the past. A few patients relied on their caregiver for reporting, highlighting a potential need for a caregiver companion app. A few expressed that the app might be more helpful if it aligned better with preexisting systems for self-management (eg, keeping real-time medication diaries). Patients identified fatigue, alternate priorities, and mental fogginess as barriers to app use, and recommended increased reminders to improve app engagement.

## Discussion

### Principal Findings

This single-arm pilot study demonstrated that MyPainPal was usable, acceptable, and showed preliminary signals of potential clinical impact on the management of cancer pain. Participants reported symptoms on the app approximately one-third of study days, which resulted in timely nurse outreach for poorly controlled symptoms. It is uncertain whether patients might have experienced similar symptom care without MyPainPal; however, in debriefing interviews, many directly credited the app with reducing barriers to symptom communication and recalled specific instances of timely clinician outreach, and reported feeling supported and validated, while gaining insights into symptom self-management. The study also identified several areas for app refinements.

While remote, PRO-based symptom monitoring interventions have proliferated in oncology [13,14] and are beginning to be applied to cancer pain [34-37], their benefits for pain have been modest [15,38]. This may be because they offer patients limited direct symptom support, tailored medication management advice, and lack the nuanced content needed to educate users and sustain engagement [39,40]. Successful pain management requires that patients and providers engage together to evaluate, understand, and treat the dynamic symptom of pain. MyPainPal addressed these foundational aspects of pain management by including robust psychoeducation on cancer pain and opioid-induced constipation, evaluating pain-related symptoms and medications, delivering tailored feedback and algorithmic self-management support, and providing care teams with nuanced symptom and medication use data in a structured format that supported outreach and population management. We found this approach to be feasible, with patients reporting good intervention usability and acceptability ratings.

In qualitative findings, patients credited MyPainPal with allowing them to gain new insights into pain self-management and promoting timely interactions with care teams, as has been seen with other PRO-based remote symptom monitoring interventions [41,42]. Approximately 1 in 5 symptom surveys triggered a clinical alert, which aligns with rates observed in other PRO interventions [43,44]. While a meaningful proportion of these alerts resulted in adjustments to patients’ pain medications before routine clinic visits, this must be balanced with the added burden to nursing staff. Future studies could evaluate varying alert thresholds or could investigate the potential of artificial intelligence to provide clinicians with management recommendations. While the exact frequency of optimal app use and symptom reporting is unclear, our findings suggest that this may depend on the severity and lability of patients’ symptoms. Interestingly, several patients described that the app helped normalize their need for opioids, reduced stigma, and may have enhanced their willingness to take opioids when needed. Opioid stigma can be a major deterrent to advanced cancer patients’ pain self-management [11,45] and may reduce clinicians’ willingness to prescribe [46-48]. Our findings suggest that standardized assessment of opioid use and education may destigmatize opioid use and potentially improve pain for patients with advanced cancer.

The study also identified several opportunities to further optimize the intervention [49], which we are implementing. Specifically, some patient-facing education was underrecognized and underused. Thus, we are creating the capability to assign educational content to bolster patient engagement and deliver behavioral evidence-based pain interventions such as cognitive behavioral therapy [50] and mindfulness. Second, we are reducing symptom survey frequencies and adding free-text entries to allow patients to communicate more nuanced information. We are also creating a feature that enables patients to log symptoms and medication use in real time to further facilitate self-monitoring and engagement. Finally, to promote scalability, we are initiating the integration of MyPainPal into the electronic medical record.

This study has several limitations. First, this single-arm pilot was unable to evaluate efficacy and its small sample size lacked statistical power to detect meaningful pre-post differences in pain; however, our encouraging findings of feasibility, acceptability, and clinical impact justify a randomized clinical trial to evaluate its impact on pain severity and pain-related health care use. Second, we tested the app in a single academic center, among a predominantly non-Hispanic White and affluent palliative care population. The generalizability of our feasibility and acceptability findings to other settings and populations is uncertain. Moreover, our population had a high symptom burden and many were near the end-of-life. While several patients suggested that MyPainPal would have been more useful earlier in their pain trajectory, this requires confirmation in a more general oncology population. Additional pilot studies are needed to evaluate the feasibility of the intervention and of a clinical trial in different clinical settings and among patients at earlier points within their care. Interestingly, a few patients relied on their caregiver to help them use the app. Caregiver-focused symptom reporting interventions have shown promise in the hospice context [51] and could be explored in future work.

### Conclusion

We found that this novel cancer pain mHealth intervention that combines symptom and medication reporting, adaptive feedback, symptom education, and care team monitoring was feasible and acceptable among patients with advanced cancer using opioids for chronic pain. Chart abstraction data and qualitative feedback suggested that patients may have benefited from the intervention through insight-building, improved care team communication, and timely medication changes. Following app refinements, randomized testing will determine its impact on relevant outcomes, including pain severity, pain interference, and pain-related acute care use.

## Appendix

Multimedia Appendix 1 Interview Guide.

Multimedia Appendix 2 Baseline and End-of-Study symptom reports.

## Abbreviations

DFCI: Dana-Farber Cancer Institute
mHealth: mobile health
EHR: electronic health record
EMA: Ecological Momentary Assessments
HIPAA: Health Insurance Portability and Accountability Act
JITAI: Just-in-Time Adaptive Intervention
NRS: Numeric Rating Scale
PANAS: Positive and Negative Affect Schedule
PRO: patient-reported outcome
REDCap: Research Electronic Data Capture
SUS: System Usability Scale

## Data Availability

The datasets generated or analyzed during this study are not publicly available due to ongoing analysis but are available from the corresponding author on reasonable request.
