# Supplementary material for: MyPainPal, a Novel mHealth App to Improve Pain in Patients With Advanced Cancer: Single-Arm Pilot Study
Source: JMIR Cancer. 2025 Dec 30;11:e79942. doi: 10.2196/79942 (PMC12811039; doi:10.2196/79942)
Supplement: Multimedia Appendix 1 [file cancer_v11i1e79942_app1.docx]

Thank you for participating in our study and using My Pain Pal. We are very excited to hear about your experience using the app, and are particularly interested in any suggestions you might have on we can make the app better.

Before we start, I want to check in with you to see if you have any more questions about the purpose of the research study and what is involved, and is it alright if I turn on the recorder?

- If no: Stop and discuss the study, elements of consent, answer questions, and obtain continuing consent to participate and be recorded prior to continuing
- If yes: Thank you, I am turning the recorder on

Our goal in creating this app is to help patients better manage their pain. As I mentioned, we want to get your feedback on the app and how helpful or unhelpful it has been to you in the past 4 weeks? Please don’t hesitate to share anything that comes to mind we are hoping to learn more about what parts of the app work and what parts we can make better!

1. To get started, could you tell us a little bit about your experience using the application?
   1. How often would you say you used it?
   2. What prompted you to use the app when you did?
   3. What did you like and dislike about the app?
   4. Was the app helpful for your pain management, and if so how?
      1. How helpful/unhelpful was the resource library?
         1. What were the parts of the resource library that you used the most and the least?
         2. Were there any aspects about your pain management you wish would have been included and wasn’t?
      2. How helpful/unhelpful was the Medication cabinet?
2. Could you tell us a little bit about what you thought about the daily surveys?
   1. How long did it usually take you to complete?
   2. Were there too many or not enough questions?
      1. If so, what would you want more or less of?
3. Let’s talk a little bit about the survey summary that you got after completing the daily surveys.
   1. How often would you say that you clicked on the recommended links in the survey summary?
   2. Were there times that you used the advice given by the application changed the way you managed your pain?
   3. How about the way you used your medications?
   4. Did you use the laxative advice given to take more or less of your laxatives?
      1. How helpful/unhelpful was the laxative advice given in the survey summary?
4. Did this change how you communicated your pain management needs to your care team?
   1. If so, less or more?
   2. Did the nurses call you ?
      1. How did that go? Helpful/unhelpful?
   3. Did this change how you reached out for support?
      1. Urgent care? ED?
   4. Did this change how you accessed opioids?
      1. Logistical changes, refills?
5. Would you say that using this app has helped you gain new insights about your pain?
   1. What behavioral skills, if any, would you say you gained from using the application?
      1. Did you use specific strategized such as activity pacing through the advice provided by the app?
   2. How helpful/unhelpful were the relaxation exercises?
   3. How helpful/unhelpful were the educational videos?
6. Lastly, would you recommend the app to a friend of yours who is trying to manage their cancer-related pain?
