# Supplementary material for: MyPainPal, a Novel mHealth App to Improve Pain in Patients With Advanced Cancer: Single-Arm Pilot Study
Source: JMIR Cancer. 2025 Dec 30;11:e79942. doi: 10.2196/79942 (PMC12811039; doi:10.2196/79942)
Supplement: Multimedia Appendix 2 [file cancer_v11i1e79942_app2.docx]

Title: A single-arm pilot study of My Pain Pal, a novel mHealth app to improve pain in patients with advanced cancer

Authors: Desiree R. Azizoddin,^1,2^ Michael Hassett^2,3^, Kris-Ann S. Anderson,^4^ Daniela Kessler^1^, Alexi A. Wright^2,3^, Madeline Gorra^3^, Benjamin S. Kematick^1^, Isaac S. Chua^1,2^, Eric Fromme^1,2^, Lida Nabati^1,2^, Douglas Brandoff^1,2^, Kate Lally,^1,2^ Susan MacIsaac,^1^ Karen Mott,^1^ William Carroll,^1^ James A. Tulsky,^1,2^ Andrea C. Enzinger^2,2^

^1^ Department of Supportive Oncology, Dana-Farber Cancer Institute, Boston, MA, USA

^2^ Harvard Medical School, Boston, MA, USA

^3^ Division of Population Sciences, Dana-Farber Cancer Institute, Boston, MA, USA

^4^Health Promotion Research Center, Stephenson Cancer Center, University of Oklahoma Health Sciences Center, Oklahoma, Oklahoma, USA

**Acknowledgements**: The study was funded by the NIH NCI R21NR017745 (Enzinger).

**Corresponding Author:**

Andrea C. Enzinger

Assistant Professor of Medicine

Harvard Medical School

Assistant Professor of Medical Oncology

Division of Population Sciences

Dana-Farber Cancer Institute

Boston, MA, USA

Email: andrea_enzinger@dfci.harvard.edu

**Supplementary Table 1**. Baseline and End-of-Study symptom reports

|  | Mean (sd) | |
| --- | --- | --- |
|  | Time 1 | Time 2 |
| BPI score total (0-10) | 4.35 (1.86) | 3.84 (1.87) |
| BPI worst pain (0-10) | 5.85 (2.39) | 5.35 (2.43) |
| BPI average pain (0-10) | 4.42 (1.81) | 4.21 (2.12) |
| BPI best pain (0-10) | 3.00 (2.18) | 2.80 (1.85) |
| BPI interference baseline (0-100) | 35.5 (14.77) | 34.55 (18.61) |
| PCS baseline (0-52) | 17.76 (12.44) | 18.25 (12.81) |
| FACT |  |  |
| Physical wellbeing  (0-28) | 14.2 (6.32) | 13.0 (6.20) |
| Social/Familial wellbeing (0-28) | 22.45 (4.62) | 22.55 (6.11) |
| Emotional wellbeing  (0-24) | 12.55 (5.12) | 9.25 (4.84) |
| Functional wellbeing (0-28) | 11.55 (4.71) | 10.80 (5.79) |
| FACT Total (0-108) | 60.75 (11.80) | 55.60 (12.69) |
| PRO-CTCAE |  |  |
| Constipation (1-5) | 1.60 (1.27) | 1.20 (1.06) |

Note: Brief Pain Inventory- Short Form (BPI-SF), Pain Catastrophizing Scale (PCS; higher scores worse), Functional Assessment of Cancer Therapy (FACT; higher scores better), PRO-CTCAE Measurement system (only constipation items were administered; 1=none, 5=very severe).
